# Inflammatory Cytokines in Cancer: Comprehensive Understanding and Clinical Progress in Gene Therapy

**DOI:** 10.3390/cells10010100

**Published:** 2021-01-08

**Authors:** Tianxia Lan, Li Chen, Xiawei Wei

**Affiliations:** 1 Laboratory of Aging Research and Cancer Drug Target, National Clinical Research Center for Geriatrics, West China Hospital, Sichuan University, No. 17, Block 3, Southern Renmin Road, Chengdu 610041, China; tianxialan77@163.com (T.L.); li_chen5525@126.com (L.C.); 2 State Key Laboratory of Biotherapy, National Clinical Research Center for Geriatrics, West China Hospital, Sichuan University, No. 17, Block 3, Southern Renmin Road, Chengdu 610041, China

**Keywords:** gene therapy, cancer therapy, inflammation, tumor microenvironment, cytokine

## Abstract

The relationship between chronic inflammation and neoplastic diseases is not fully understood. The inflammatory microenvironment of a tumor is an intricate network that consists of numerous types of cells, cytokines, enzymes and signaling pathways. Recent evidence shows that the crucial components of cancer-related inflammation are involved in a coordinated system to influence the development of cancer, which may shed light on the development of potential anticancer therapies. Since the last century, considerable effort has been devoted to developing gene therapies for life-threatening diseases. When it comes to modulating the inflammatory microenvironment for cancer therapy, inflammatory cytokines are the most efficient targets. In this manuscript, we provide a comprehensive review of the relationship between inflammation and cancer development, especially focusing on inflammatory cytokines. We also summarize the clinical trials for gene therapy targeting inflammatory cytokines for cancer treatment. Future perspectives concerned with new gene-editing technology and novel gene delivery systems are finally provided.

## 1. Introduction

Inflammation is a complicated process that functions as a biological response to harmful stimuli and may trigger common symptoms such as fever, swelling and pain [1]. It has been reported that inflammation is associated with the development of many diseases such as metabolic disorders, obesity, autoimmune diseases and neoplastic diseases [2,3]. Among them, cancer is the deadliest disease for human beings. According to data provided by the International Agency for Research on Cancer, about 9.5 million people died from cancer in 2018 [4]. Therefore, a deeper insight into the links between inflammation and cancer is important, and it is worthwhile to comprehensively evaluate the mediators of cancer-related inflammation that could potentially be implemented into anticancer therapy. Interestingly, inflammation possesses both pro- and anti-tumor features. As part of the immune response, inflammation can activate immune cells and induce the production of inflammatory proteins such as cytokines and enzymes to inhibit the growth of tumors. Thus, multiple biotherapies have been designed to potentiate such antitumor effects for the treatment of cancer. However, inflammation may contribute to the initiation, promotion and metastasis of cancer [5]. It has been revealed that around 25% of cancer cases are related to chronic inflammation [6]. Importantly, previous clinical experiences have demonstrated that nonspecific anti-inflammatory drugs such as aspirin have manifested anticancer potencies [7]. Thus, properly manipulating anti-cancer inflammation or pro-cancer inflammation has been considered as a promising strategy for the treatment of cancer. Notably, in clinical or preclinical settings, many researchers are currently trying to achieve the modulation of cancer-related inflammation using gene therapies. Hence, it is important to comprehensively analyze clinical progress in such gene therapies to evaluate their therapeutic prospects.

The concept of gene therapy is developed under the theory that the correction of cancer-related mutations can control or inhibit the growth of cancer cells and improve patients’ outcomes. The field of gene therapy is underpinned by the development of pathology, immunology as well as material science and nanotechnology. The gene delivery system, including viral and nonviral vectors, also determines its clinical safety and efficiency. Armed by the deeper understanding of mechanisms and pathways underlying the development of cancer, the concept has been further extended to treatments that inhibit or produce mediators of cancer.

In this review, we introduce the critical inflammatory mediators in cancer development and highlight the clinical trials of gene therapy targeting the inflammatory microenvironment in cancer treatment through the years.

## 2. Inflammation and Cancer

### 2.1. Inflammation and Cancer Development

For a long time, inflammation has been thought to be a double-edged sword. Although the appropriate stimulation of inflammation can induce the elevation of immune response against cancer, studies have also suggested that inflammatory factors are involved in tumorigenic processes [8].

Inflammatory processes participate in the initiation, promotion and metastasis of cancer through various mechanisms [8]. The initiation of a tumor requires a series of genetic mutations and epigenetic modifications that lead to the activation of the tumorigenic pathway as well as the loss of tumor suppression. In an inflammatory microenvironment, macrophages and neutrophils are active producers of reactive oxygen species (ROS) as well as reactive nitrogen (RNS), both of which can cause DNA damage, which is closely associated with the initiation of the tumorigenesis process [9]. Furthermore, DNA damage can be induced by cytokines. For example, Gronke et al. have shown that IL-22 can activate DNA damage response by regulating the expression of a series of genes [10]. Moreover, epigenetic modifications that upregulate the expression of oncogenes or downregulate the expression of tumor-suppressor genes are found to be orchestrated by inflammatory cytokines [11]. Thus, the components of cancer-related inflammation provide a friendly environment for cancer initiation.

Inflammation partakes in the promotion of cancer. Many studies have demonstrated that various inflammatory factors are involved in the cellular or molecular processes that facilitate tumor growth and progression [8]. In particular, NF-κB plays an important role in the manipulation of the tumor microenvironment (TME). It controls the expression of cytokines that regulate cell growth and migration [12,13]. By collaborating with cytokines, transcription factors such as NF-κB establish an intricate system that allows the substantial expansion of tumors. Mediators such as cells and other functional proteins contribute to angiogenesis, the inhibition of immune response, the regulation of metabolism and other supportive activities for cancer promotion [14,15,16]. It would be valuable to further explore the prospects of targeting these pathways for cancer therapies. In addition, inflammation is an important contributor to the metastasis of tumors. Tumor metastasis requires the migration and seeding of cancer cells, survival of the early metastatic colony and establishment of new TME [17]. Cellular programs such as epithelial-to-mesenchymal transition (EMT) also have a positive effect on the mobility of cancer cells, thereby facilitating invasion and metastasis [18]. Considering the inefficiency of metastatic processes [19], every supportive factor for the accomplishment of metastasis needs to be deeply understood, as blocking them may be an effective strategy to improve the life expectancy of patients with metastatic diseases. Particularly, inflammatory cytokines are important orchestrators of cancer–inflammation interactions, influence multiple aspects of cancer metastasis. For instance, it has been shown that IL-11 could enhance the growth of the most invasive subset of breast cancer cells [20]. Additionally, TNFs are capable of activating the expression of the transcription factor that can induce EMT [21].

Together, inflammation and the cancer microenvironment modulated by inflammatory response influence initiation, promotion and metastasis through an intricate network. A wide range of inflammatory factors, including cytokines, transcriptional factors, immune cells and stromal cells, are fundamental components of this system.

### 2.2. Cytokines in Cancer-Related Inflammation

**Figure 1:**
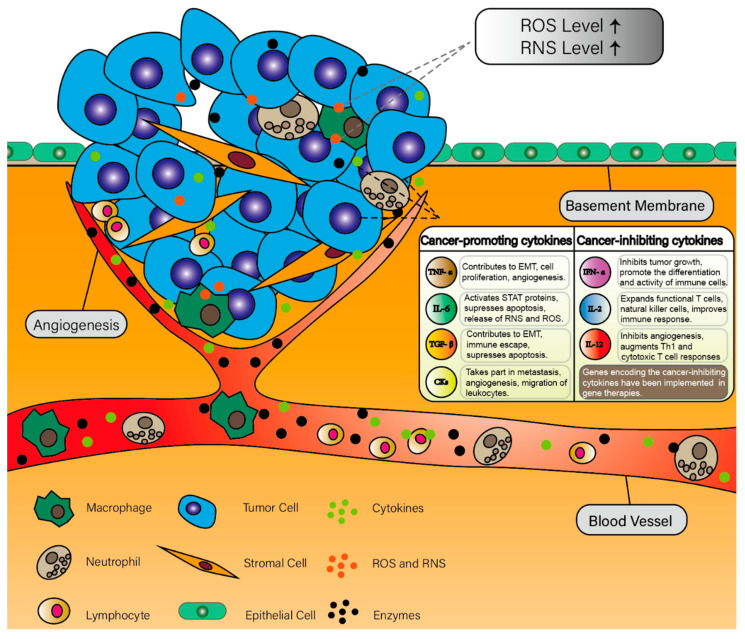
Critical inflammatory mediators in the tumor microenvironment.

Cytokines are a group of functional proteins secreted from the immune system. They were initially described as modulators of immune response and inflammation [22]. However, an increasing number of studies found that the elevations of some types of cytokines are associated with the induction and progression of tumors [23]. Hence, the roles of these cancer-related cytokines have gained considerable attention [24]. These cytokines that promote or enhance cancer development are involved in a coordinated system (Figure 1).

Although the mechanisms governing some pathways have been illustrated by in vitro studies or animal studies, many cytokines were found to be elevated in patients with cancer or tumor microenvironments [25,26]. Thus, it is of vital importance to fully explore how these cytokines interact with tumor cells and the tumor microenvironment, as a deep understanding of the underlying mechanisms and pathways could shed substantial light on the development of potential anticancer therapies.

#### 2.2.1. Interleukins

Interleukin 6 (IL-6), mainly secreted by monocytes, conducts its functions by binding to IL-6 receptors [27]. In the context of inflammation, the production of IL-6 by immune cells is usually a result of infections and tissue injuries [28]. The pro-oncogenesis effects of IL-6 have been demonstrated in various cancer types, including lung cancer, breast cancer and colorectal cancer, among others [29,30,31]. IL-6 can act with proteins in the STAT family to assist tumorigenic processes [32,33]. Moreover, the cytokine is associated with the inhibition of apoptotic programs as well as the release of ROS and RNS [26,34]. Although targeting IL-6 is considered to be a promising anticancer treatment, it has yet to be integrated into anticancer strategies as a gene therapy. However, gene therapy modulating Il-6 has been studied in noncancer diseases such as hepatic failure and infections [35]. The prospect of targeting IL-6 in cancer settings still needs to be explored by more future studies.

On the contrary, some other inflammatory interleukins such as interleukin 2 (IL-2) and interleukin 12 (IL-12) possess anticancer effects. The clinical efficacy of gene-edited lymphocyte transfer has been verified in lung cancer patients. The overall response rate (ORR) was significantly higher in the group treated with IL-2 gene therapy. Moreover, a substantial decrease in tumor size was also observed in the cell therapy group. The reported toxicity of this treatment was minimal. Hence, the clinical results indicate that this method of cancer gene therapy is safe and possibly efficacious against pleural effusions caused by lung cancer [36]. Furthermore, IL-12 is capable of activating cytotoxic immune cells [37], thus making it a useful substance for the induction of immune response against cancer [38].

#### 2.2.2. Tumor Necrosis Factor Alpha

Tumor necrosis factor alpha (TNF-α) is an inflammatory cytokine that participates in the regulation of a variety of signaling processes [39]. By attaching to TNF-α R-1 and TNF-α R-2 [26], the cytokine impacts tumor development via multiple mechanisms, such as contributing to EMT, boosting the cell proliferation rate and accelerating angiogenesis, among others [40,41,42]. The aberrant expression of TNF-α was found in a variety of neoplastic diseases, including prostate cancer, ovarian cancer, liver cancer and breast cancer [43,44,45,46]. The antitumor activities of TNF-α have been leveraged in cancer treatments [47]. Interestingly, the cytokine has also been found to exert cancer-promoting roles [48]. A recent study revealed that TNF-α may contribute to the migration of tumor cells by regulating prion protein levels [49].

#### 2.2.3. Transforming Growth Factor Beta

Transforming growth factor beta (TGF-β) is also a well-documented pleiotropic cytokine that has been found in many oncogenic pathways [34,50]. TGF-β produced by inflammatory cells, including neutrophils and macrophages, plays a substantial role in tumor initiation and progression [51], and studies have shown that blockage of TGF-β significantly increased the effects of anticancer treatments [52,53]. Similar to IL-6 and TNF-α, TGF-β is associated with a wide range of tumor-inductive or cancer-supportive mechanisms, such as EMT, immune escape, the formation of blood vessels, as well as the suppression of apoptotic pathways [52,54]. In breast cancer, TGF-β enhances the vasculature within TME by regulating the expression of VEGF and MCP-1 [55]. Furthermore, The elevated expression level of TGF-β has been found in epithelial ovarian cancer cells and prostate cancer TME [56,57].

#### 2.2.4. Chemokines

Chemokines (CKs) regulate the activation and migration of multiple types of cells. In the context of inflammation, the migration of leukocytes to inflammatory sites is mediated by CKs [58]. This type of cytokine is also closely associated with cancer progression, and metastasis as well as angiogenesis mediated by chemokines have been broadly studied [59,60]. Many anticancer approaches have been designed to target chemokines for inhibiting metastasis and angiogenesis [61,62,63,64]. The gene of C-C motif chemokine ligand 21 (CCL-21) has been introduced into dendritic cells with adenovirus, and these genetically edited dendritic cells could be injected into patients as an antitumor vaccine. In a phase I trial (NCT01574222), patients with non-small-cell lung cancer received such a vaccine to enhance the immune response against tumors. The results show that this strategy successfully evoked the antigen-specific immune response and substantial infiltration of CD8+ T cells [65].

#### 2.2.5. Interferons

Interferons (IFNs) are a group of cytokines crucial for inflammatory processes. IFNs regulate the molecular, cellular and physiological processes that govern inflammatory responses. So far, three types of IFNs (type I, type II, type III) have been uncovered, classified based on their structural characteristics and the specific receptors to which they bind [66]. In cancer, IFNs can affect metabolism and the proliferation of cancer cells via a wide spectrum of molecular pathways. They are also inducers of apoptosis [67]. Type I interferons, for example, possess strong tumor inhibitory ability. It has been recently reported that IFN-I induces the activation of signal transducers and activators of transcription 3 (STAT-3) and activates a series of downstream signals to achieve the inhibition of tumor progression [68]. Equipped with the accumulating understanding of the antitumor functions of IFNs, some type I and type II IFNs have already been directly or indirectly applied to the development of anticancer therapies. Type I interferons such as IFN-α have been developed into Food and Drug Administration (FDA)-approved drugs for the treatment of cancer or hepatitis C (e.g., Intron^®^ A and Roferon^®^ A). The gene encoding IFN-α has also been inserted into viral vectors for developing antitumor gene therapies.

## 3. Clinical Studies of Gene Therapies Targeting Inflammatory Cytokines in Cancer

**Figure 2:**
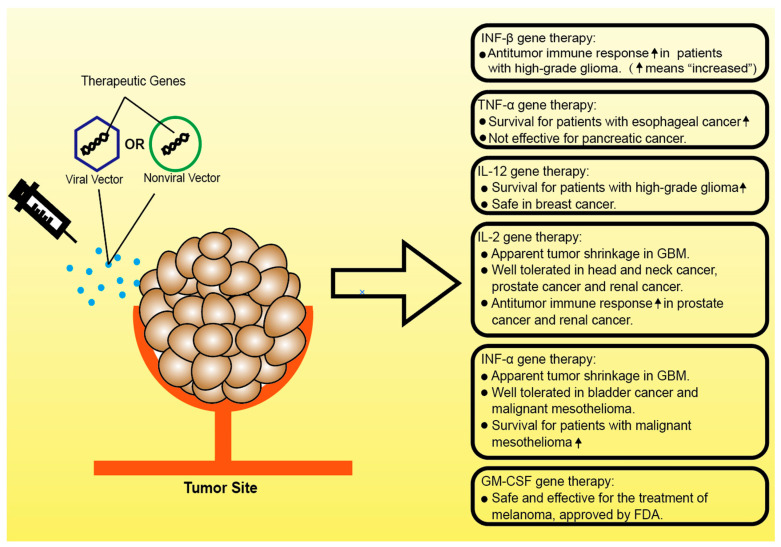
Clinical progress on inflammatory cytokine gene therapies.

In the context of cancer treatment, the manipulation of cancer-related inflammation is capable of improving patient outcomes [7]. While anti-inflammation drugs such as nonsteroidal anti-inflammatory drugs (NSAIDs) reduce pain, decrease fever and suppress cancer progression when administered in a high dose, proinflammation drugs such as interleukins are also being considered as effective cancer immunotherapies. However, with respect to the gene therapy, previous or current clinical studies are largely focused on utilizing the antitumor effects of proinflammatory proteins, more specifically, inflammatory cytokines (Figure 2).

At present, the biological roles of many inflammatory cytokines have been deeply explored. Some of these cytokines have been considered ideal targets for antitumor therapies, and others are thought to be potent immunomodulatory factors that can be used for treating cancer. Considerable effort has gone into investigating the efficacy and safety of antibodies that inhibit the tumor-promoting pathways regulated by inflammatory functional proteins [69,70,71,72]. However, the therapeutic value of targeted gene therapies that modulate cancer-related inflammation has yet to be comprehensively evaluated. Important previous or ongoing clinical trials with critical inflammatory cytokine gene therapies are summarized in Table 1.

### 3.1. Gene Therapies Based on TNF-α

As an important inflammatory cytokine, TNF-α has been used in cancer therapies for decades. Many strategies, including antibody–drug conjugates, cell therapy and fusion proteins, have been investigated in preclinical models or patients with different types of cancers [84,85,86,87]. Gene therapies have also been assessed in preclinical and clinical settings [88,89]. Although off-target effects are a major concern for gene therapies, the local administration of drugs and recently more advanced gene-editing tools significantly increased the convenience and specificity of gene editing.

Although it is clear that TNF-α has both pro- and anticancer effects, clinical studies of gene therapies have mainly focused on exploiting the anticancer side of the cytokine. In a phase II study (NCT00051480), in combination with chemoradiotherapy, the TNF-α gene was intratumorally delivered to patients with locally advanced esophageal cancer with adenovirus vectors. Although the clinical outcome of the phase II trial was not published, the results from a phase I study of the drug showed a good safety profile and the prolonged survival of patients. In all 24 enrolled patients, the median overall survival (mOS) was 48.7 months, and the five-year survival rate was 41% [90]. The same drug has also been evaluated in patients with prostate cancer, pancreatic cancer, rectal cancer, head and neck cancer and melanoma (NCT01048151; NCT00496535; NCT00051467; NCT00137878; NCT00261404). Among them, the clinical study for locally advanced pancreatic cancer reached phase III. However, the therapy failed to effectively prolong the length of patients’ survival. According to the published data, no significant difference was found between the patients who received gene therapy and standard of care (SOC) and the patients who only received SOC (10.0 months vs. 10.0 months). The patients in the SOC-only group showed longer progression-free survival than the gene therapy group [89]. As a consequence, the therapy was not approved for the treatment of pancreatic cancer.

In addition, one ongoing clinical trial was registered to assess the safety of the adenovirus containing genes encoding TNF-α and IL-2 proteins. The phase I study of the drug was initiated in February 2020 (NCT04217473). The trial is expected to be completed in 2021, and the sponsor recently announced that two patients had already passed the primary safety endpoint.

### 3.2. Gene Therapies Based on IL-12

IL-12 is not only an important immunomodulating cytokine but also a strong mediator of cancer development. It exerts proinflammatory functions by activating cytotoxic immune cells [91], thus making it an important cytokine in the antitumor response of the immune system [92]. Gene therapies designed to leverage the functions of IL-12 have been evaluated in clinical trials. In a phase I study (NCT02026271), during the surgical resection process, a human IL-12 gene was delivered into patients with glioblastoma multiforme or anaplastic oligoastrocytoma. For the management of toxicity, the production of IL-12 was controlled by the oral activator (veledimex), which was administered before and after the operation to regulate the expression of human IL-12. The safety profile was acceptable, and median overall survival (mOS) of 12.7 months was observed. Notably, in a subgroup of the trial where patients additionally received more than 20 mg of dexamethasone in 14 days, mOS was 16.7 months [36]. The 16.7 months of survival is a very encouraging number for the treatment of patients with recurrent high-grade glioma because the disease is generally considered hard to treat. The significant effects of adding dexamethasone to the therapy are worth further exploring.

IL-12 gene therapy has also been used in combination with chemotherapy and pembrolizumab for the treatment of triple-negative breast cancer. In a phase II study (NCT04095689), docetaxel, doxorubicin, cyclophosphamide, pan-nitric oxide synthase (NOS) inhibitor NG-monomethyl-L-arginine and pembrolizumab plus IL-12 gene therapy were administered to patients with early triple-negative breast cancer, as the phase I trial of the trial showed the safety of the strategy. The trial is estimated to commence in December 2020 and is still recruiting participants. The design of this combination of drugs is theoretically effective for the treatment of breast cancer, as adding pembrolizumab to the chemotherapy regimen has been shown to increase the efficacy of treatment, and IL-12 gene therapy is supposed to facilitate the antitumor immune response.

### 3.3. Gene Therapies Based on IL-2

IL-2 is also a strong proinflammatory cytokine [37]. Importantly, it is described as the “first effective immunotherapy” against tumors [93]. The IL-2 gene has also been integrated into the virus for the delivery of cytokines in order to strengthen the antitumor immune response. Previously, Colombo, et al. described that patients with glioblastoma multiforme (GBM) were intratumorally injected with a retroviral vector carrying the IL-2 gene and the thymidine kinase gene of herpes simplex virus type 1 (HSV-TK). HSV-TK is considered a “suicide” gene as it regulates the programs that induce cell death. The IL-2 gene was added to enhance the antitumor effects mediated by HSV-TK. In the clinical study, four patients were administered the virus. Two patients died from tumor growth 5 and 12 months after the treatment, and the remaining two developed stabilized diseases. Apparent tumor shrinkage was observed in one of the patients who died from pulmonary embolism [94]. These results show that combining the IL-2 and HSV-TK genes in the virus has antitumor effects, but the safety and the efficacy still need to be validated in larger clinical studies.

In a more recent clinical trial, a plasmid was used as the vector of the IL-2 gene, and the nonviral gene therapy was designed for the treatment of head and neck cancer. In the phase I study, patients were intratumorally injected with the drug in escalating doses. The results suggest that the gene therapy was well tolerated [73]. A similar strategy has also been used in treating prostate cancer. In another phase I study, a plasmid carrying the IL-2 gene was contained in a DNA/lipid complex for therapeutic delivery. The patients were intraprostatically injected with the drug weekly for two weeks. After the treatment, no grade 3 or 4 adverse events were reported, indicating that the drug was well tolerated by the patients. Furthermore, evidence for a stronger antitumor immune response such as the increased infiltration of T cells and accelerated proliferation of lymphocytes was observed [75], suggesting the considerable immunomodulatory effect of the drug.

Later, data from another similar phase I clinical study of IL-2 gene therapy were published. In this study, the IL-2 gene was carried by an adenovirus vector for treating localized prostate cancer. A total of 12 patients were intraprostatically injected with the virus, and a grade 3 adverse event was only observed in one patient. No grade 4 adverse event was reported [74]. The antitumor response induced by the treatment was comparable to that induced by the previous clinical trial using plasmid-mediated IL-2 delivery (T cells and lymphocytes infiltration).

Lastly, IL-2 based gene therapy has also been used for treating renal cancer. The drug is basically composed of a plasmid containing the gene for IL-2. For each patient, the drug was injected into multiple sites of the tumor. After four cycles of the administration, no grade 3 or 4 treatment related adverse events were observed in all 31 patients. The ORR was 10%, and 23% of patients achieved stable disease. The mOS was 11 months. The additional evaluation of CD8+ lymphocyte infiltration demonstrated the induction of a strengthened immune response [76].

### 3.4. Gene Therapies Based on IFN-α

While it has been reported that IFN-α partakes in the activation of inflammasomes by promoting the expression of caspase-11 [95], it is considered a group of effective regulators of an antitumor immune response. Genetically edited viruses that express interferons have been developed into drugs for the treatment of cancer. Recombinant adenoviruses that lost the ability to replicate were modified to express interferon α2b (IFN-α2b). In a phase II study (NCT01687244), 43 patients with bladder cancer received the virus, formulated by a supportive polyamide surfactant. The drug was administered intravesically. The results showed that 35% of the patients achieved recurrence-free survival, with no observations of treatment-related adverse events of grade 4–5 in any of the patients [77]. The outcomes of this phase II study indicate that it will be a promising replacement for patients unable or unwilling to undergo radical cystectomy.

In an earlier phase I study (NCT01119664), the IFN-α2b-expressing adenovirus was administered along with celecoxib in patients with malignant mesothelioma, followed by chemotherapy. In a pilot clinical study, 40 patients received the IFN-α2b-expressing adenovirus, celecoxib, in combination with first- or second-line chemotherapies. After the treatment, the ORR of all patients was 25%, the disease control rate (DCR) was 88% and the two-year survival rate 32%. The results suggest that the treatment is safe in patients and also prolongs the survival rate compared to historical control [78].

### 3.5. Gene Therapies Based on IFN-β

Interferon β (IFN-β) has been shown to manifest both pro- and anti-inflammatory features [96]. It is currently being used as an immunomodulator for the treatment of cancers. Gene therapy based on interferon β (IFN-β) has also been evaluated in clinical studies to access the effects of the overexpression of IFN-β on neoplastic diseases. In a phase I study, IFN-β genes carried by a cationic liposome were injected into patients with high-grade glioma. The clinical outcomes indicate that the administration of the drug substantially improves the antitumor response of the immune system by inducing necrosis as well as the infiltrations of macrophages and CD8+ lymphocytes [79]. Moreover, the IFN-β gene has been introduced into adenovirus for the treatment of pleural malignancies and mesothelioma (NCT00299962; NCT01119664), and both trials have been completed.

### 3.6. Gene Therapies Based on GM-CSF

Granulocyte-macrophage colony-stimulating factor (GM-CSF) enhances the inflammatory response by promoting the activation of immune cells [97]. Furthermore, GM-CSF is being used as an immunostimulant in cancer therapies [98]. In 1998, Michael, et al. reported that they had used a GM-CSF-encoding virus as a gene therapy to treat patients with melanoma. Over the course of the six-week treatment, the virus carrying a passenger gene was intratumorally injected into patients every two weeks. The treatment was safe, and only limited and mild adverse events were observed. The transcribed mRNA of virally encoded GM-CSF was observed in all patients. Although the concentrations of blood cells or immune cells in patients’ blood were not found to be influenced by the treatment, in all seven patients who received the treatment, three had a mixed response and one patient had a partial response, indicating the considerable efficacy of the therapeutic virus [80].

More recently, the oncolytic virus was genetically modified to express GM-CSF for the treatment of cancer, and the efficacy and safety were evaluated by clinical trials. In the phase I study, patients with head and neck cancer, breast cancer, melanoma and gastrointestinal cancer were intratumorally injected with the therapeutic oncolytic virus as a subsequent-line treatment. The drug was well tolerated, the oncolytic virus successfully induced the enhancing of antitumoral immune response in patients and tumor necrosis was observed in more than half of the patients [81]. In the following phase II study, fifty patients with unresectable metastatic melanoma were recruited. They were intratumorally injected with the genetically edited oncolytic virus, and the safety and efficacy of the treatment were further evaluated. According to the published data, the virus was safely tolerated in patients, with an overall response rate (ORR) of 26%. Fifty-eight percent of the patients survived for one year after the treatment, and the two-year survival rate was 52% [82]. These results stress the prospect of this biological drug for the effective management of melanoma, which was successfully granted for the phase III study. A total of 436 patients were enrolled in the phase III trial. Two-thirds of them were assigned to the oncolytic virus group, and one-third were injected with the GM-CSF protein. ORR and median overall survival (mOS) were assessed for the ultimate evaluation of the therapy. The results show that patients in the oncolytic virus group had better ORR (26.4% vs. 5.7%) and mOS (23.3 months vs. 18.9 months). Furthermore, grade 3 or 4 adverse events were only found in 2% of patients in the oncolytic virus group [83], suggesting that the GM-CSF-based gene therapy is an ideal approach for improving the outcome of melanoma treatment. In October 2015, the genetically manipulated oncolytic virus product was approved by the FDA for melanoma gene therapy, namely T-VEC [99].

## 4. Future Perspectives and Ongoing Preclinical Studies

In recent years, the dawn of gene therapy has come due to the thriving development of gene-editing technologies and biomedical material science. Boosted by the considerable progress made in gene-editing technologies such as clustered regularly interspaced short palindromic repeats (CRISPR) and gene editor techniques, gene therapies can be conducted in a more convenient and precise manner [100]. Although there is substantial concern surrounding the possible side effects, including off-target effects and the introduction of new mutations, the advantages of gene therapy such as one-time dosage and high plasticity make it one of the most promising new medical developments of this century [101]. Moreover, in the fields of material science and nanotechnology, the first nanoparticle gene therapeutic, which is a lipid complex containing small interfering RNA (siRNA), was approved by the US Food and Drug Administration (FDA) for the treatment of a rare disease in 2018. [102] Thus, here, we summarize some representative studies of gene therapy targeting the cancer inflammatory environment using either CRISPR or novel nonviral vectors, which might be promising to move into a clinical trial in the future.

### 4.1. CRISPR Targeting Cancer Inflammatory Environment

The CRISPR/Cas9 system is a potent tool for gene editing. CRISPR/Cas9 could potentially be used in the treatment of a wide range of disorders caused by DNA mutations. Many CRISPR-based cancer therapies aim to modify the gene in tumor cells to inhibit growth and proliferation or reduce the immune-escaping capacity. Nanoparticles containing the CRISPR/Cas9 system were recently delivered into mice with glioblastoma (GBM) to disrupt the expression of the polo-like kinase 1 (PLK1) gene. The protein encoded by the PLK1 gene is currently believed to be a potent target for cancer therapy [103]. In the study, the in vivo tumor model was established by injecting GBM cells to the hippocampus of mice, and the nanoparticles containing the CRISPR/Cas9 system were intratumorally injected into mice. As a result, the treatment significantly inhibited tumor growth and increased the survival rate of mice [104]. This study has consequently shed light on the encouraging prospects of CRISPR-based gene therapy.

It has been reported that a research group achieved the in vivo gene-editing of macrophages in mice. Their results show that the guide RNA successfully bound to the target gene, Cas9 expression was induced by a macrophage-specific promoter and the knockout of Nnt1 was validated by the reduced expression of proteins encoded by the target gene. In addition, the Cas9 expressions of other cells such as T cells, B cells and neutrophils were negligible, suggesting that the CRISPR/Ca9-mediated gene editing in this study is specific to macrophages [105]. In another study, the CRISPR/Cas9 system was used to block the activation of the NLRP3 inflammasome of macrophages in mice, and the delivery of guide RNA and Cas9 was assisted by cationic lipid-assisted nanoparticles. The Cas9 gene was specifically expressed in microphages and disrupted the expression of the NLRP3 inflammasome, leading to the meditation of inflammatory consequences such as peritonitis and septic shock in mice [106].

The success of the precise and efficient gene editing of inflammatory factors mediated by CRISPR/Cas9 in animal models reveals the substantial promise of achieving more optimistic outcomes in clinical trials. Future studies may focus on combining such advanced gene-editing tools with well-established anticancer therapies to bring new hopes for patients.

### 4.2. Novel Nonviral Vectors

In the past, viral vectors were most frequently used for the delivery of gene therapies. However, in clinical practices, there is much concern with respect to the safety surrounding viral vectors. The capacity of viral vectors to contain therapeutic genes is limited. Such disadvantages elicit the quick development of nonviral vectors such as hydrogels and nanoparticles. If properly made, such vectors could theoretically contain the gene of any length. Furthermore, the manufacturing of nonviral vectors is cost-effective [107,108].

In the context of gene therapy, nonviral vectors have been used in clinical or preclinical studies for the delivery of therapeutic genes. Cationic copolymers have been used as vectors for genes encoding cytokines (e.g., GM-CSF, IL-2) [76,109]. In preclinical settings, the polyethylenimine-polyethylene glycol copolymer has been broadly studied. For example, Irina et al. complexed the cDNA of GM-CSF and HSVtk with a cationic copolymer for the delivery of gene therapy. Data suggest that therapeutic genes were expressed in mice, confirmed by the detection of the active GM-CSF and HSVtk protein. The in vivo experiments showed that the treatment significantly reduced the tumor volume of mice. Notably, the similar “cytokine gene + suicide gene” combination has been evaluated in a previously described clinical study [94] where adenovirus was used as a vector. Thus, future clinical studies assessing the clinical value of gene therapy carried out by the polyethylenimine-polyethylene glycol copolymer would provide important data for the comparison of viral and nonviral vectors.

Additionally, nonviral vectors are widely used for gene therapies mediated by CRISPR/Cas9. Ling et al. complexed the CRISPR/Cas9 system with a fluorinated polymer to knock out the gene encoding human mutT homolog 1 (MTH1) in vivo. The gene was to be overexpressed in a broad range of cancers [110]. Recent evidence suggests that the upregulation of MTH1 expression is correlated with cancer-related inflammation [111]. In the in vivo study targeting MTH1 in ovarian cancer, the efficiency of fluorinated polymer delivery was found to be superior to traditional nonviral systems such as Lipofectamine 2000 and Lipofectamine 3000, and the growth of tumors was effectively inhibited in mice [112].

Although nonviral vectors are also associated with disadvantages such as relatively low transfection efficiency and a short duration of time of the therapeutic gene expression, they still hold the promise to become effective gene-transfer systems with more affordable prices.

## 5. Conclusions

With increasing evidence linking inflammation with cancer development, more critical mediators within cancer-related inflammation can be revealed. The mechanism underlying the pathways regulated by these mediators also needs to be further explored. Inflammatory cytokines are involved in multiple cancer development processes, which makes them ideal antitumor agents or targets. Given that much research has been conducted evaluating the safety and efficacy of different therapies regarding inflammatory cytokines, no significant progress has been demonstrated in terms of clinical values. Armed with the rise of new technologies such as CRISPR/Cas9 and nonviral vectors in the 21st century, it is hopeful that gene therapies based on inflammatory cytokines could provide miracles in the fight against cancer.

## Figures and Tables

**Table 1:** Important previous or ongoing clinical trials with inflammatory cytokine gene therapies.

	Cytokine Genes	Transfer Vector	Cancer Applications	Phase	Key Publication or clinicaltrials.gov No.
TNF	TNF-α	Adenovirus	Prostate cancer	II	NCT01048151
	TNF-α	Adenovirus	Pancreatic cancer	III	NCT00051467
	TNF-α	Adenovirus	Esophagus cancer	II	NCT00051480
	TNF-α	Adenovirus	Head and neck cancer	I/II	NCT00496535
	TNF-α	Adenovirus	Rectal cancer	II	NCT00137878
	TNF-α	Adenovirus	Melanoma	II	NCT00261404
	TNF-α and IL-2	Adenovirus	Melanoma	I	NCT04217473
IL	IL-12	Adenovirus	Glioma	I	NCT02026271 [36]
	IL-12	Adenovirus	Breast cancer	II	NCT04095689
	IL-2	Plasmid	Head and neck cancer	I	[73]
	IL-2	Adenovirus	Prostate cancer	I	[74]
	IL-2	Plasmid	Prostate cancer	I	[75]
	IL-2	Cationic lipid	Renal cancer	I/II	[76]
IFN	IFN-α2b	Adenovirus	Bladder cancer	II	[77] NCT01687244
	IFN-α2b	Adenovirus	Mesothelioma	I	[78] NCT01119664
	IFN-β	Adenovirus	Glioma	I	[79]
	IFN-β	Adenovirus	Pleural malignancies	I	NCT00299962
GM-CSF	GM-CSF	Vaccinia virus	Melanoma	Not reported	[80]
	GM-CSF	Oncolytic virus	Head and neck cancer; breast cancer; melanoma; gastrointestinal cancer	I	[81]
	GM-CSF	Oncolytic virus	Melanoma	II	[82]
	GM-CSF	Oncolytic virus	Melanoma	III	[83]

## Data Availability

No new data were created or analyzed in this study. Data sharing is not applicable to this article.
